# Dataset on the influence of gas-to-liquid biosludge on arid soil properties and growth performance of alfalfa

**DOI:** 10.1016/j.dib.2019.105074

**Published:** 2020-01-03

**Authors:** Reginald B. Kogbara, Wubulikasimu Yiming, Srinath R. Iyengar, Udeogu C. Onwusogh, Karim Youssef, Marwa Al-Ansary, Parilakathoottu A. Sunifar, Dhruv Arora, Osman A.E. Abdalla, Hayel M. Al-Wawi

**Affiliations:** a Mechanical Engineering Program, Texas A&M University at Qatar, P.O. Box 23874, Education City, Doha, Qatar; b Qatar Shell Research & Technology Center, QSTP LLC, Doha, Qatar; c Department of Agricultural Research, Ministry of Municipality & Environment, Doha, Qatar

**Keywords:** Alfalfa, Arid soil, Fresh weight biomass, Gas-to-liquid biosludge, Mineralogical composition, Plant growth parameters, Porosity, Soil conditioner

## Abstract

The dataset presented here is related to our research article entitled “Effect of gas-to-liquid biosludge on soil properties and alfalfa yields in an arid soil” [1]. It relates to selected performance parameters of alfalfa grown in an arid soil amended with five different (0.75–12%) gas-to-liquid biosludge contents, and selected properties of the soil determined using several material characterization techniques. A detailed description of the raw data relating to figures on alfalfa performance parameters such as fresh biomass weight, plant height, the number of tillers, and biomass elemental content in the companion article is provided alongside additional data on the number of days to flowering. The underlying data for leachate from the soil and underlying spectra and diffractograms for the proton nuclear magnetic resonance (^1^H-NMR) and X-ray diffraction (XRD) data, respectively, shown in the companion article are presented. These show changes in the pore structure characteristics and the mineralogical composition of the soil, soil-fertilizer, soil-biosludge, and soil-compost mixtures tested over time. Additional data showing the effect of the amendments on the bulk and particle densities of the soil is presented. The dataset demonstrates the influence of the industrial biosludge on arid soil properties and alfalfa yields (Kogbara et al., [1]).

**Table:** Specifications Table

Subject	*Environmental Science*
Specific subject area	*Waste Management and Disposal*
Type of data	*Table, Image, Figure, Raw data*
How data were acquired	*Field measurements, Magritek* 2 MHz *nuclear magnetic resonance (NMR) rock core analyzer, Rigaku Ultima IV multipurpose X-ray diffractometer, standard test methods for determination of metals and anions in water samples, and standard test methods for laboratory determination of density of soil specimens.*
Data format	*Raw, analyzed and calculated*
Parameters for data collection	*Direct field measurements of plant performance parameters, NMR measurements of cumulative porosity, T*_*2*_*distribution and T*_*2*_*log-mean, XRD patterns collected at 2theta (2θ) angle from 3 to 80 degrees with a step size of 0.01 degree and scanning speed of 0.5°/min.*
Description of data collection	*Alfalfa performance parameters such as aboveground fresh biomass weight was collected at about* 5 cm *above ground level during each cut. The plant height, the number of tillers and the number of days to flowering were determined by direct measurement, counting and field observation of the plants, respectively. Leachate characteristics and soil density parameters were determined using standard methods for water and soil analysis. Pore structure characteristics and mineralogical composition were determined using the aforementioned NMR and XRD instruments.*
Data source location	*Doha, Qatar.*
Data accessibility	*Data is within this article.*
Related research article	R.B. Kogbara, W. Yiming, S.R. Iyengar, O.A.E. Abdalla, H.M. Al-Wawi, U.C. Onwusogh, K. Youssef, M. Al-Ansary, P.A. Sunifar, D. Arora. Effect of gas-to-liquid biosludge on soil properties and alfalfa yields in an arid soil. *Journal of Cleaner Production*, https://doi.org/10.1016/j.jclepro.2019.119524 [1].

**Table:** 

**Value of the Data** • The dataset is useful as it provides information on the effect of biosludge from the wastewater treatment plant of a gas-to-liquid (GTL) plant on soil properties and growth performance of a forage crop, alfalfa, in a region with challenging soil and climatic conditions. • The dataset provides insights, which can be used by researchers, agricultural scientists, civil/environmental engineers, and environmental management practitioners to understand how the amendment of arid soils with GTL biosludge influences soil properties and affect plant growth. • The dataset can serve as a starting point for the planning of field trials related to the evaluation of the growth performance of different (forage or industrial) crops in biosludge-amended arid soils. • Additionally, the data can assist young researchers in understanding how to employ the advanced material characterization equipment used here to determine soil properties such as pore structure characteristics and mineralogical composition.

## 1 Data description

The raw data relating to figures on alfalfa performance parameters such as fresh biomass weight, plant height and the number of tillers in the companion article [1] are provided in Table 1, Table 2, Table 3 respectively. These are presented for each of the three replicates in a given treatment. In contrast, these data were calculated and simply reported as the mean and standard deviation in the companion article [1]. Additional data not shown in the companion article on the number of days to flowering is provided in Table 4. Table 5, Table 6, Table 7 show the raw data on the elemental content of the plant biomass at each of the first, second and third cuts, which were summarily presented as the averages of the three cuts in the companion article [1].

**Table 1:** Detailed data on fresh biomass weight of alfalfa grown in soil, soil-fertilizer, soil-biosludge, and soil-compost mixtures.

Fresh biomass weight (g/plant)															
Treatment	First Cut					Second Cut					Third Cut				
	Rep. I	Rep. II	Rep. III	Mean	Std. dev.	Rep. I	Rep. II	Rep. III	Mean	Std. dev.	Rep. I	Rep. II	Rep. III	Mean	Std. dev.
Soil (C1)	190	216	167	191.0	24.5	176	216	194	195.3	20.0	80	81	80	80.3	0.6
Soil + NPK + Urea (C2)	291	305	214	270.0	49.0	165	264	152	193.7	61.3	105	98	95	99.3	5.1
Soil + 3% C (C3)	167	189	201	185.7	17.2	174	221	342	245.7	86.7	88	115	102	101.7	13.5
Soil + 0.75% BS (E1)	258	272	264	264.7	7.0	331	314	299	314.7	16.0	99	92	105	98.7	6.5
Soil + 1.5% BS (E2)	298	223	239	253.3	39.5	325	259	334	306.0	41.0	87	100	88	91.7	7.2
Soil + 3% BS (E3)	202	148	194	181.3	29.1	366	233	245	281.3	73.6	97	91	107	98.3	8.1
Soil + 6% BS (E4)	165	190	129	161.3	30.7	307	270	211	262.7	48.4	78	79	78	78.3	0.6
Soil + 12% BS (E5)	95	86	53	78.0	22.1	290	217	215	240.7	42.7	74	84	86	81.3	6.4

Rep.: Replicate, Std. dev.: Standard deviation, NPK: 20-20-20 NPK fertilizer, C: Compost, BS: Biosludge.

**Table 2:** Detailed data on plant height for alfalfa grown in soil, soil-fertilizer, soil-biosludge, and soil-compost mixtures.

Plant height (cm)															
Treatment	First Cut					Second Cut					Third Cut				
	Rep. I	Rep. II	Rep. III	Mean	Std. dev.	Rep. I	Rep. II	Rep. III	Mean	Std. dev.	Rep. I	Rep. II	Rep. III	Mean	Std. dev.
Soil (C1)	45	49	48	47.3	2.1	62	74	58	64.7	8.3	51	57	56	54.7	3.2
Soil + NPK + Urea (C2)	64	53	44	53.7	10.0	74	73	64	70.3	5.5	58	74	81	71.0	11.8
Soil + 3% C (C3)	43	50	41	44.7	4.7	69	72	78	73.0	4.6	63	105	95	87.7	21.9
Soil + 0.75% BS (E1)	85	62	51	66.0	17.3	92	79	67	79.3	12.5	91	95	89	91.7	3.1
Soil + 1.5% BS (E2)	63	50	61	58.0	7.0	78	66	76	73.3	6.4	79	69	84	77.3	7.6
Soil + 3% BS (E3)	59	49	53	53.7	5.0	79	69	73	73.7	5.0	79	77	65	73.7	7.6
Soil + 6% BS (E4)	62	34	33	43.0	16.5	75	71	63	69.7	6.1	74	59	80	71.0	10.8
Soil + 12% BS (E5)	33	41	15	29.7	13.3	55	61	67	61.0	6.0	60	58	62	60.0	2.0

Rep.: Replicate, Std. dev.: Standard deviation, NPK: 20-20-20 NPK fertilizer, C: Compost, BS: Biosludge.

**Table 3:** Detailed data on the number of tillers for alfalfa grown in soil, soil-fertilizer, soil-biosludge, and soil-compost mixtures.

Number of tillers															
Treatment	First Cut					Second Cut					Third Cut				
	Rep. I	Rep. II	Rep. III	Mean	Std. dev.	Rep. I	Rep. II	Rep. III	Mean	Std. dev.	Rep. I	Rep. II	Rep. III	Mean	Std. dev.
Soil (C1)	5	5	5	5	0	6	6	6	6	0	5	6	6	6	1
Soil + NPK + Urea (C2)	4	5	4	4	1	6	7	6	6	1	6	7	6	6	1
Soil + 3% C (C3)	4	5	3	4	1	5	7	7	6	1	6	6	5	6	1
Soil + 0.75% BS (E1)	5	6	4	5	1	7	7	6	7	1	7	7	7	7	0
Soil + 1.5% BS (E2)	5	5	5	5	0	6	6	6	6	0	6	6	7	6	1
Soil + 3% BS (E3)	4	4	5	4	1	8	6	6	7	1	7	7	6	7	1
Soil + 6% BS (E4)	6	5	3	5	2	7	6	5	6	1	6	6	6	6	0
Soil + 12% BS (E5)	3	4	2	3	1	5	6	7	6	1	5	5	5	5	0

Rep.: Replicate, Std. dev.: Standard deviation, NPK: 20-20-20 NPK fertilizer, C: Compost, BS: Biosludge.

**Table 4:** Data on the number of days to flowering for alfalfa grown in soil, soil-fertilizer, soil-biosludge, and soil-compost mixtures.

Number of days to flowering															
Treatment	First Cut					Second Cut					Third Cut				
	Rep. I	Rep. II	Rep. III	Mean	Std. dev.	Rep. I	Rep. II	Rep. III	Mean	Std. dev.	Rep. I	Rep. II	Rep. III	Mean	Std. dev.
Soil (C1)	–	–	–	–	–	44	51	47	47.3	3.5	13	14	14	13.7	0.6
Soil + NPK + Urea (C2)	–	–	–	–	–	44	44	44	44.0	0.0	13	14	14	13.7	0.6
Soil + 3% C (C3)	–	–	–	–	–	44	45	47	45.3	1.5	14	13	12	13.0	1.0
Soil + 0.75% BS (E1)	79	79	79	79	0	51	44	44	46.3	4.0	13	10	14	12.3	2.1
Soil + 1.5% BS (E2)	–	–	–	–	–	44	48	49	47.0	2.6	13	10	10	11.0	1.7
Soil + 3% BS (E3)	–	–	–	–	–	44	44	44	44.0	0.0	14	13	13	13.3	0.6
Soil + 6% BS (E4)	–	–	–	–	–	44	46	44	44.7	1.2	12	13	10	11.7	1.5
Soil + 12% BS (E5)	–	–	–	–	–	47	44	48	46.3	2.1	11	11	15	12.3	2.3

Rep.: Replicate, Std. dev.: Standard deviation, NPK: 20-20-20 NPK fertilizer, C: Compost, BS: Biosludge.

**Table 5:** Detailed data on the elemental content of the plant biomass during the first cut.

Treatment	Sample	Na	K	Ca	Mg	Al	Cr	Mn	Fe	Ni	Cu	Zn
Soil (C1)	Rep. I	5320	22320	26010	3790	146.6	–	72.7	362.4	6.8	6.5	110.1
	Rep. 2	5440	22340	29720	4300	118.3	–	152.9	350.0	–	8.8	95.4
	Rep. 3	8090	19780	26270	3960	116.4	–	65.0	305.5	–	7.1	53.6
	Average	6283.3	21480.0	27333.3	4016.7	127.1	–	96.8	339.3	6.8	7.5	86.4
Soil + NPK + Urea (C2)	Rep. I	9540	17060	30970	4420	113.7	–	74.2	414.9	–	36.4	48.7
	Rep. 2	7040	18750	27430	4410	137.4	–	182.5	324.0	1.3	34.0	60.6
	Rep. 3	8920	20360	31320	4810	157.8	–	78.8	413.6	0.8	7.9	82.4
	Average	8500.0	18723.3	29906.7	4546.7	136.3	–	111.8	384.2	1.1	26.1	63.9
Soil + 3% C (C3)	Rep. I	8660	25790	28060	4010	133.9	–	68.0	316.3	–	12.8	60.2
	Rep. 2	10290	27920	26960	4690	168.0	–	64.7	335.0	–	22.0	83.2
	Rep. 3	12590	27290	24320	5560	159.0	9.4	58.6	338.4	20.8	58.1	57.0
	Average	10513.3	27000.0	26446.7	4753.3	153.6	9.4	63.7	329.9	20.8	31.0	66.8
Soil + 0.75% BS (E1)	Rep. I	7070	24100	28930	5340	118.7	5.0	81.8	333.9	6.4	18.4	78.8
	Rep. 2	8280	21890	28640	4580	147.7	–	71.5	420.8	115.0	67.0	82.7
	Rep. 3	10140	25690	24540	5380	109.9	–	55.5	273.9	50.5	59.7	43.5
	Average	8496.7	23893.3	27370.0	5100.0	125.4	5.0	69.6	342.9	57.3	48.3	68.4
Soil + 1.5% BS (E2)	Rep. I	8920	18250	29660	4290	140.2	–	82.1	316.6	4.2	46.8	34.5
	Rep. 2	9490	23130	29450	4780	166.4	–	73.8	368.4	0.5	61.9	65.0
	Rep. 3	8790	29540	39610	6570	198.2	–	153.9	501.7	0.3	32.7	116.8
	Average	9066.7	23640.0	32906.7	5213.3	168.3	–	103.3	395.6	1.7	47.1	72.1
Soil + 3% BS (E3)	Rep. I	9730	14890	34400	5760	164.4	1.4	90.1	449.2	6.1	53.0	76.0
	Rep. 2	9170	31120	30250	5070	121.7	1.1	75.1	333.4	7.3	22.2	69.4
	Rep. 3	13690	22870	30610	5730	344.1	1.8	84.3	550.3	7.3	25.5	100.9
	Average	10863.3	22960.0	31753.3	5520.0	210.1	1.4	83.2	444.3	6.9	33.6	82.1
Soil + 6% BS (E4)	Rep. I	9320	24600	29060	5200	131.5	–	114.6	275.3	–	28.6	80.1
	Rep. 2	11460	21500	30880	5250	210.5	–	78.3	386.3	0.8	26.1	58.7
	Rep. 3	8670	19070	29790	4610	123.2	1.0	81.1	286.1	0.2	18.6	25.6
	Average	9816.7	21723.3	29910.0	5020.0	155.1	1.0	91.4	315.9	0.5	24.4	54.8
Soil + 12% BS (E5)	Rep. I	11350	20750	29710	5040	235.7	–	75.6	383.3	–	12.3	99.5
	Rep. 2	10690	23280	30630	4980	237.2	–	80.3	400.0	–	8.4	76.3
	Rep. 3	15170	33800	26640	6380	324.0	0.7	61.8	416.7	2.7	8.9	46.2
	Average	12403.3	25943.3	28993.3	5466.7	265.6	0.7	72.6	400.0	2.7	9.9	74.0

**Table 6:** Detailed data on the elemental content of the plant biomass during the second cut.

Treatment	Sample	Na	K	Ca	Mg	Al	Cr	Mn	Fe	Ni	Cu	Zn
Soil (C1)	Rep. I	3760	28220	15870	3180	278.4	–	72.7	362.4	6.8	6.5	110.1
	Rep. 2	4900	27820	18590	3540	189.5	–	152.9	350.0	ND	8.8	95.4
	Rep. 3	5540	27900	16700	4910	341.5	–	65.0	305.5	ND	7.1	53.6
	Average	4733.3	27980.0	17053.3	3876.7	269.8	–	96.8	339.3	6.8	7.5	86.4
Soil + NPK + Urea (C2)	Rep. I	6360	26990	16060	4650	272.9	–	74.2	414.9	ND	36.4	48.7
	Rep. 2	4580	26830	16420	3540	253.1	–	182.5	324.0	1.3	34.0	60.6
	Rep. 3	4500	24930	14320	3940	217.4	–	78.8	413.6	0.8	7.9	82.4
	Average	5146.7	26250.0	15600.0	4043.3	247.8	–	111.8	384.2	1.1	26.1	63.9
Soil + 3% C (C3)	Rep. I	5200	29880	17770	4190	263.6	–	68.0	316.3	ND	12.8	60.2
	Rep. 2	6240	25870	21580	5090	372.7	–	64.7	335.0	ND	22.0	83.2
	Rep. 3	8090	27260	19040	5190	285.0	9.4	58.6	338.4	20.8	58.1	57.0
	Average	6510.0	27670.0	19463.3	4823.3	307.1	9.4	63.7	329.9	20.8	31.0	66.8
Soil + 0.75% BS (E1)	Rep. I	6560	26350	19490	4340	415.3	5.0	81.8	333.9	6.4	18.4	78.8
	Rep. 2	4420	27630	14740	3520	216.4	–	71.5	420.8	115.0	67.0	82.7
	Rep. 3	6930	32590	16160	5760	358.8	–	55.5	273.9	50.5	59.7	43.5
	Average	5970.0	28856.7	16796.7	4540.0	330.2	5.0	69.6	342.9	57.3	48.3	68.4
Soil + 1.5% BS (E2)	Rep. I	4760	26170	15890	3980	381.0	–	82.1	316.6	4.2	46.8	34.5
	Rep. 2	5110	25480	14120	4030	259.2	–	73.8	368.4	0.5	61.9	65.0
	Rep. 3	5660	28660	22190	4200	249.8	–	153.9	501.7	0.3	32.7	116.8
	Average	5176.7	26770.0	17400.0	4070.0	296.7	–	103.3	395.6	1.7	47.1	72.1
Soil + 3% BS (E3)	Rep. I	5990	24070	23360	5270	204.9	1.4	90.1	449.2	6.1	53.0	76.0
	Rep. 2	6340	28280	23200	5010	157.3	1.1	75.1	333.4	7.3	22.2	69.4
	Rep. 3	5410	28770	17510	4370	283.5	1.8	84.3	550.3	7.3	25.5	100.9
	Average	5913.3	27040.0	21356.7	4883.3	215.2	1.4	83.2	444.3	6.9	33.6	82.1
Soil + 6% BS (E4)	Rep. I	5810	28740	18830	4010	250.5	–	119.6	242.6	6.8	47.1	122.1
	Rep. 2	4540	28200	13790	4060	251.6	–	40.5	102.3	10.7	66.3	84.7
	Rep. 3	5590	26980	16010	4140	277.2	0.6	45.6	204.7	7.9	10.8	45.6
	Average	5313.3	27973.3	16210.0	4070.0	259.8	0.6	68.6	183.2	8.5	41.4	84.1
Soil + 12% BS (E5)	Rep. I	6850	24420	26100	5550	157.6	–	56.9	220.9	3.0	16.4	63.0
	Rep. 2	5980	26030	15400	3880	313.1	1.2	37.8	261.9	19.3	24.9	96.1
	Rep. 3	7080	27920	15710	4740	221.3	0.3	37.4	136.9	3.7	7.7	58.8
	Average	6636.7	26123.3	19070.0	4723.3	230.7	0.7	44.0	206.6	8.7	16.3	72.6

**Table 7:** Detailed data on the elemental content of the plant biomass during the third cut.

Treatment	Sample	Na	K	Ca	Mg	Al	Cr	Mn	Fe	Ni	Cu	Zn
Soil (C1)	Rep. I	14270	29760	18610	5543	223.7	0.6	82.7	314.7	11.4	12.3	73.0
	Rep. 2	18790	26550	21610	5891	216.3	0.8	248.6	396.7	6.6	6.1	89.2
	Rep. 3	15690	28690	18950	6559	229.6	1.0	64.0	348.1	4.0	9.0	104.2
	Average	16250.0	28333.3	19723.3	5997.7	223.2	0.8	131.8	353.2	7.4	9.1	88.8
Soil + NPK + Urea (C2)	Rep. I	12970	28530	18080	6468	221.7	0.8	59.9	314.5	2.9	6.3	106.8
	Rep. 2	19190	30870	19190	5503	222.9	0.7	201.3	328.5	14.7	16.9	81.5
	Rep. 3	12940	28000	13990	6386	198.6	0.5	52.2	224.0	1.9	6.4	79.9
	Average	15033.3	29133.3	17086.7	6119.0	214.4	0.7	104.4	289.0	6.5	9.9	89.4
Soil + 3% C (C3)	Rep. I	19370	27590	18880	6199	143.6	0.8	48.7	235.3	3.5	8.1	91.5
	Rep. 2	13330	26050	16460	5868	190.7	0.6	53.6	268.5	1.3	6.8	106.6
	Rep. 3	18720	36600	14850	6605	194.9	0.7	47.3	204.8	1.5	7.1	83.1
	Average	17140.0	30080.0	16730.0	6224.0	176.4	0.7	49.8	236.2	2.1	7.4	93.7
Soil + 0.75% BS (E1)	Rep. I	15510	29390	18520	5825	181.5	1.3	78.5	281.0	1.0	12.1	119.3
	Rep. 2	13760	30380	18410	6150	227.4	0.8	64.7	304.2	5.8	8.1	108.6
	Rep. 3	21520	43880	14180	7322	161.8	0.7	52.7	132.7	1.3	8.8	88.4
	Average	16930.0	34550.0	17036.7	6432.3	190.2	0.9	65.3	239.3	2.7	9.7	105.4
Soil + 1.5% BS (E2)	Rep. I	8353	29700	14890	5796	217.2	0.5	84.1	250.3	3.8	7.2	65.7
	Rep. 2	19230	27370	21130	5711	477.3	2.9	210.3	645.1	3.5	4.1	67.4
	Rep. 3	13810	26930	12730	6096	185.3	0.4	50.0	184.5	2.3	9.2	72.3
	Average	13797.7	28000.0	16250.0	5867.7	293.3	1.2	114.8	360.0	3.2	6.8	68.4
Soil + 3% BS (E3)	Rep. I	18190	25970	23800	7660	214.2	0.9	50.6	371.9	6.6	2.0	17.0
	Rep. 2	19600	28120	22560	7605	274.4	1.0	60.3	413.3	3.5	3.2	40.7
	Rep. 3	12260	29600	17950	6847	139.9	0.1	79.3	206.7	3.5	5.3	69.2
	Average	16683.3	27896.7	21436.7	7370.7	209.5	0.7	63.4	330.6	4.5	3.5	42.3
Soil + 6% BS (E4)	Rep. I	20170	28870	17530	6105	189.1	0.5	123.6	251.3	ND	3.4	76.9
	Rep. 2	13160	29780	17100	6747	279.3	1.0	55.3	345.9	3.2	7.1	115.5
	Rep. 3	11400	28560	17180	6514	2421.0	16.7	103.9	2895.0	11.3	10.5	60.5
	Average	14910.0	29070.0	17270.0	6455.3	963.1	6.1	94.3	1164.1	7.3	7.0	84.3
Soil + 12% BS (E5)	Rep. I	21670	28150	29820	7441	971.5	5.6	72.5	1388.0	6.4	2.2	22.8
	Rep. 2	15320	24940	14980	5637	334.8	1.5	48.0	324.0	ND	4.2	97.4
	Rep. 3	23660	35840	12050	6640	315.5	1.5	51.8	333.8	ND	5.2	43.3
	Average	20216.7	29643.3	18950.0	6572.7	540.6	2.9	57.4	681.9	6.4	3.9	54.5

**Fig. 1:**
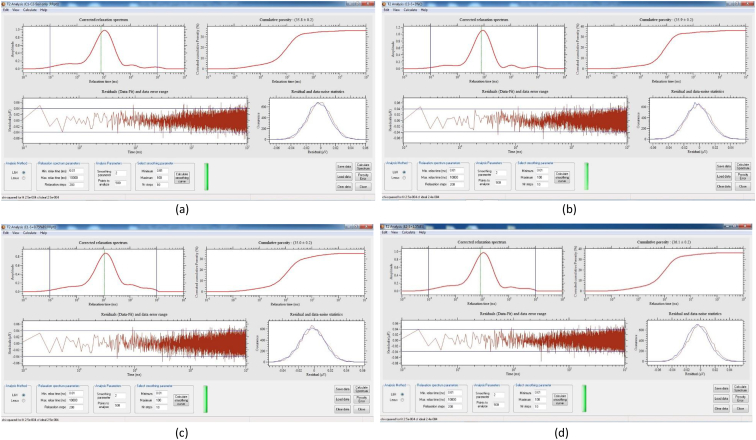
Screenshot of NMR T_2_ relaxation analysis showing (from left to right in each image) the underlying spectra for the T_2_ distribution (proxy for pore size distribution) and cumulative porosity data, residuals (data-fit) and data error range, and residual and data-noise statistics for the (a) Soil, (b) Soil + 3% compost, (c) Soil + 0.75% biosludge, and (d) Soil + 1.5% biosludge mixtures before planting. *Note: The Soil and Soil + NPK + Urea treatments are similar before planting. The green vertical dotted line in the T*_*2*_*distribution spectrum indicates the T*_*2*_*log-mean (proxy for mean pore size).*

**Fig. 2:**
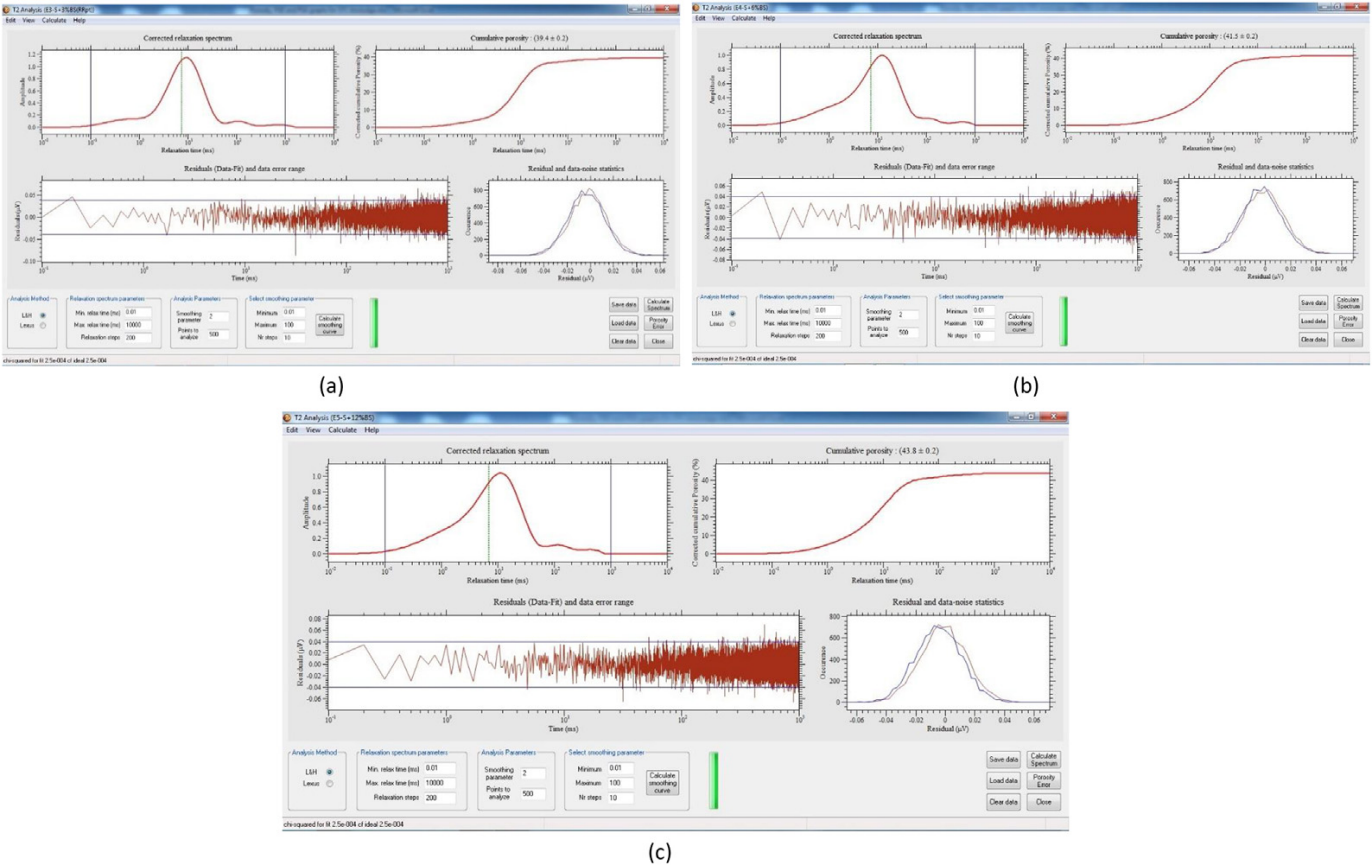
Screenshot of NMR T_2_ relaxation analysis showing (from left to right in each image) the underlying spectra for the T_2_ distribution (proxy for pore size distribution) and cumulative porosity data, residuals (data-fit) and data error range, and residual and data-noise statistics for the (a) Soil + 3% biosludge, (b) Soil + 6% biosludge, and (c) Soil + 12% biosludge mixtures before planting. *Note: The green vertical dotted line in the T*_*2*_*distribution spectrum indicates the T*_*2*_*log-mean (proxy for mean pore size).*

**Fig. 3:**
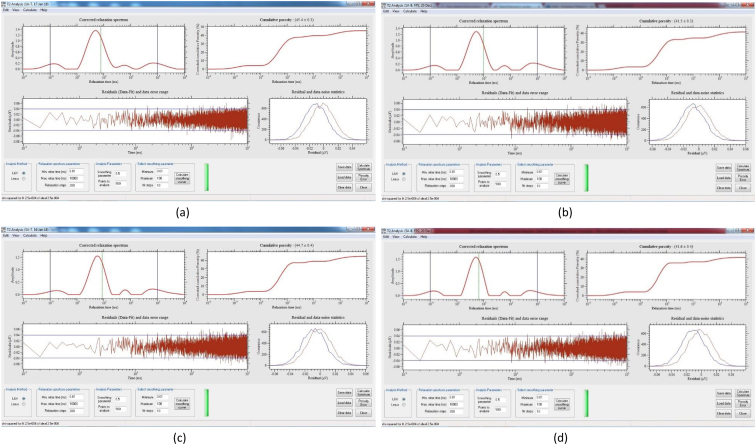
Screenshot of NMR T_2_ relaxation analysis showing (from left to right in each image) the underlying spectra for the T_2_ distribution (proxy for pore size distribution) and cumulative porosity data, residuals (data-fit) and data error range, and residual and data-noise statistics for the top and bottom layers, respectively, of the (a) and (b) Soil, and (c) and (d) Soil + NPK + Urea mixtures at the final-growth stage. *Note: The green vertical dotted line in the T*_*2*_*distribution spectrum indicates the T*_*2*_*log-mean (proxy for mean pore size).*

**Fig. 4:**
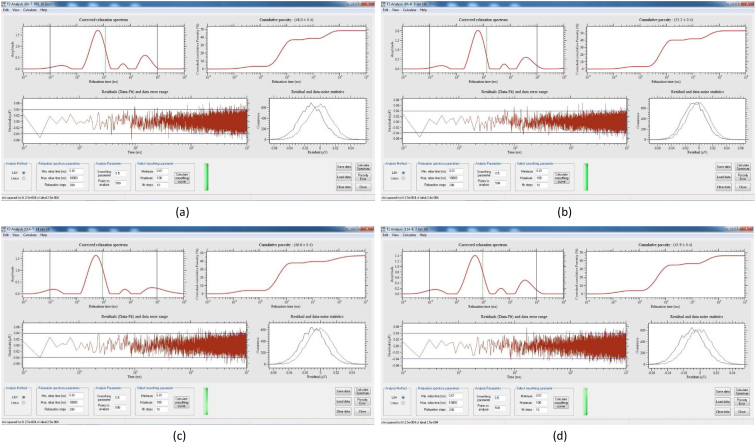
Screenshot of NMR T_2_ relaxation analysis showing (from left to right in each image) the underlying spectra for the T_2_ distribution (proxy for pore size distribution) and cumulative porosity data, residuals (data-fit) and data error range, and residual and data-noise statistics for the top and bottom layers, respectively, of the (a) and (b) Soil + 3% compost, and (c) and (d) Soil + 0.75% biosludge mixtures at the final-growth stage. *Note: The green vertical dotted line in the T*_*2*_*distribution spectrum indicates the T*_*2*_*log-mean (proxy for mean pore size).*

**Fig. 5:**
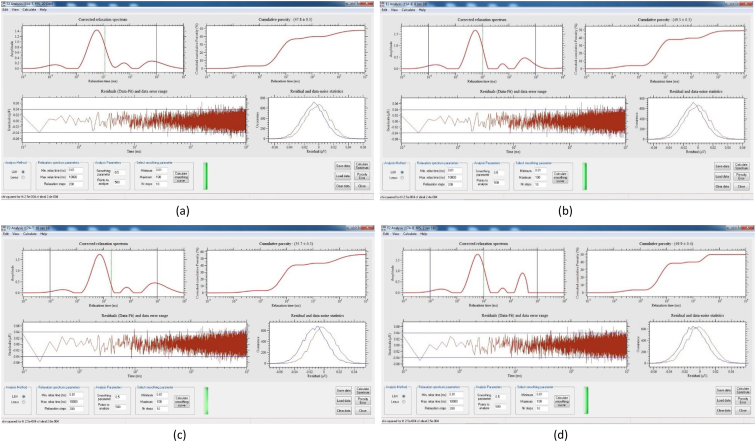
Screenshot of NMR T_2_ relaxation analysis showing (from left to right in each image) the underlying spectra for the T_2_ distribution (proxy for pore size distribution) and cumulative porosity data, residuals (data-fit) and data error range, and residual and data-noise statistics for the top and bottom layers, respectively, of the (a) and (b) Soil + 1.5% biosludge, and (c) and (d) Soil + 3% biosludge mixtures at the final-growth stage. *Note: The green vertical dotted line in the T*_*2*_*distribution spectrum indicates the T*_*2*_*log-mean (proxy for mean pore size).*

**Fig. 6:**
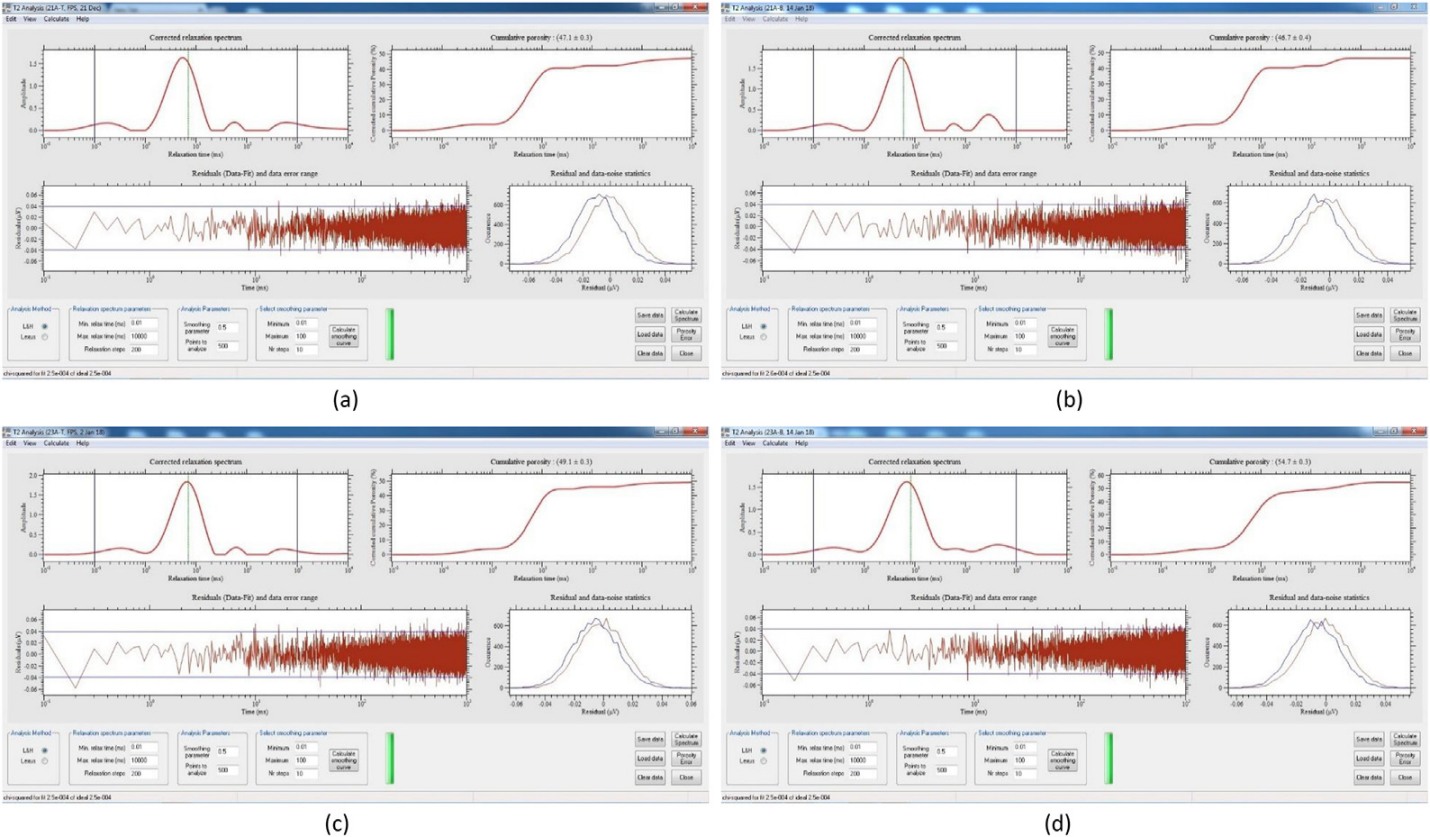
Screenshot of NMR T_2_ relaxation analysis showing (from left to right in each image) the underlying spectra for the T_2_ distribution (proxy for pore size distribution) and cumulative porosity data, residuals (data-fit) and data error range, and residual and data-noise statistics for the top and bottom layers, respectively, of the (a) and (b) Soil + 6% biosludge, and (c) and (d) Soil + 12% biosludge mixtures at the final-growth stage. *Note: The green vertical dotted line in the T*_*2*_*distribution spectrum indicates the T*_*2*_*log-mean (proxy for mean pore size).*

**Fig. 7:**
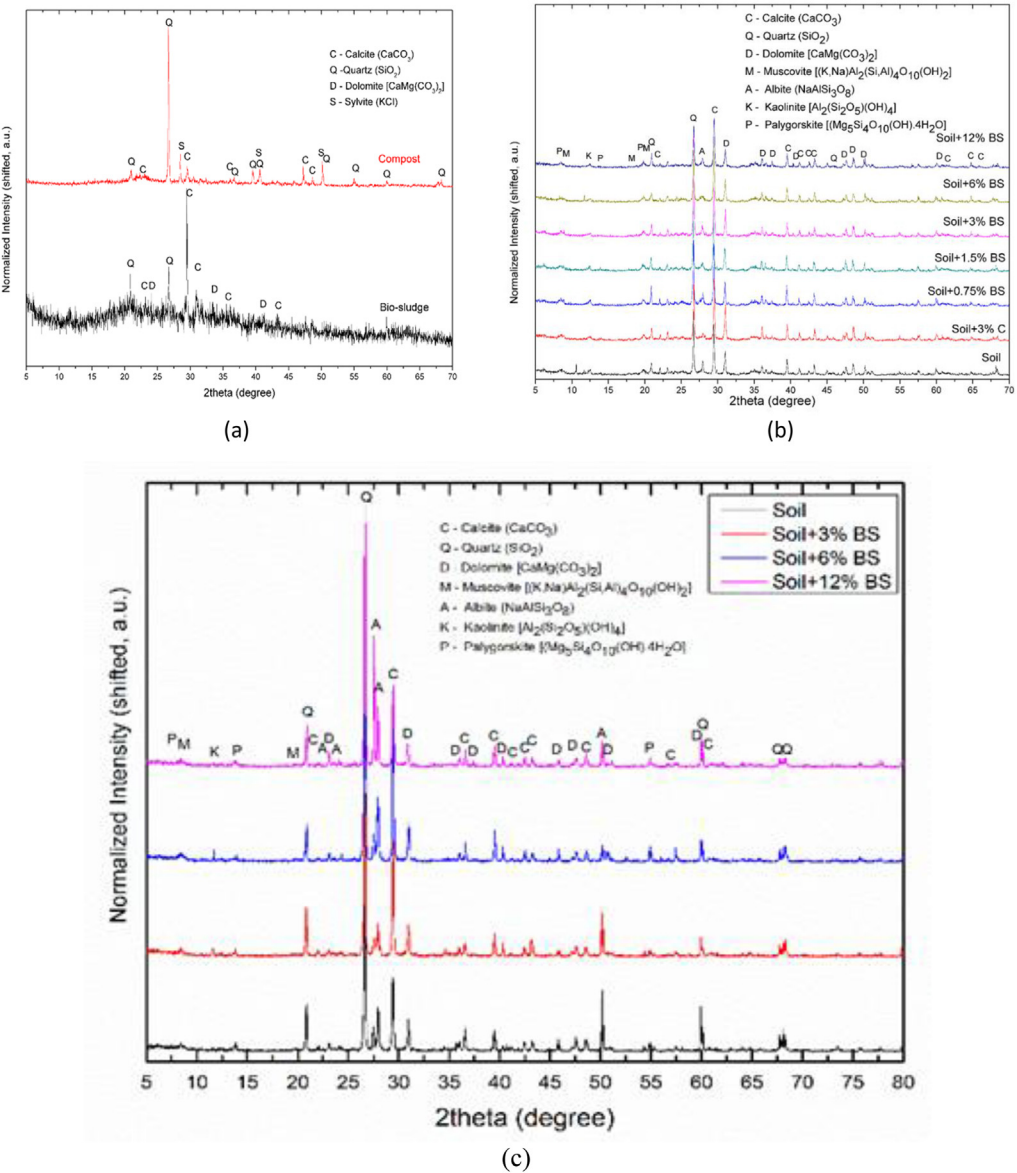
XRD diffratograms showing the mineralogical composition of the (a) biosludge, and (b) soil, soil-biosludge and soil-compost mixtures, before planting, and (c) selected treatments at the final-growth stage. *Note: BS – Biosludge, C – Compost. The systematic change of mineral weight percentage with increasing biosludge content at the initial (before planting) stage is not apparent since all treatments contained various amounts of amorphous materials. Hence, the analysis at the final growth stage focused on selected treatments, namely, soil, and soil with 3, 6 and 12% biosludge contents.*

**Fig. 8:**
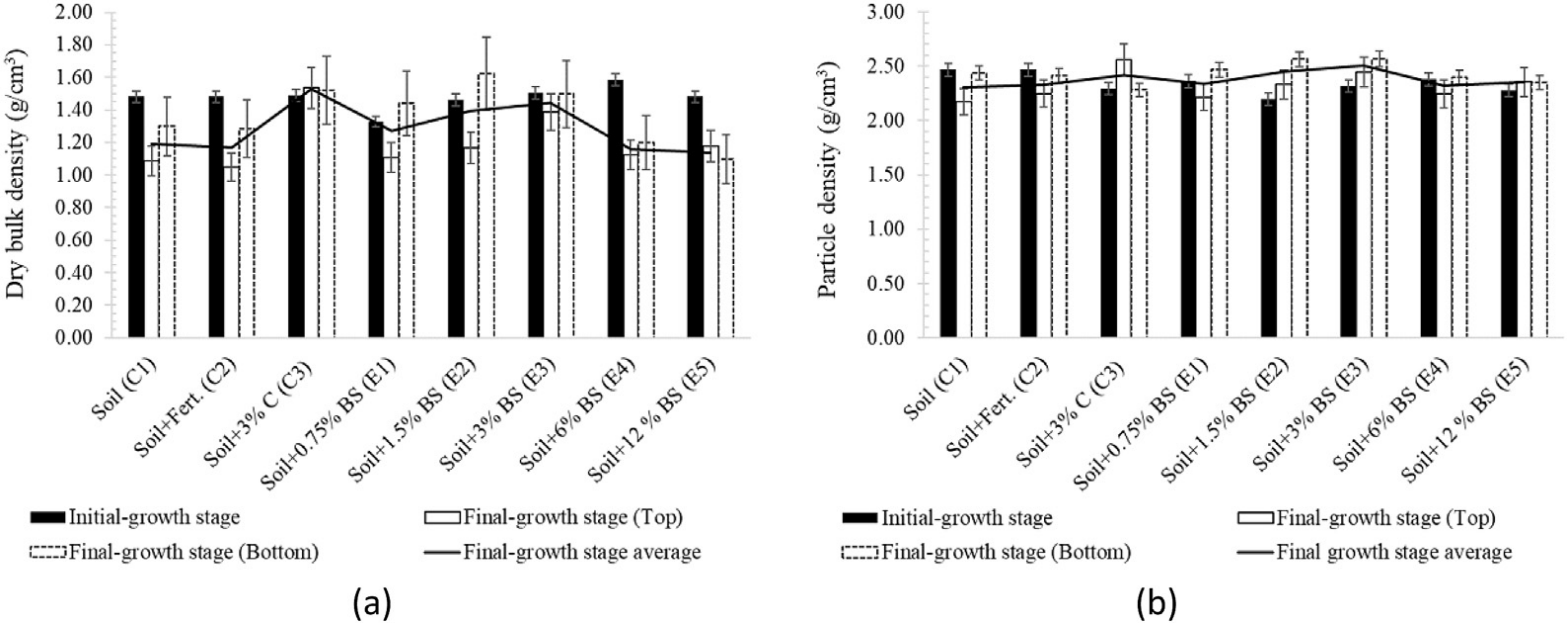
Density analysis of the different treatments at the initial (before planting) and final-growth stages in terms of (a) dry bulk density, and (b) particle density. *Note: BS – Biosludge, C – Compost, Fert. – Fertilizer (NPK + Urea). The dry bulk densities of biosludge and compost are 0.66 and 0.65 g/cm3, respectively. The particle densities of biosludge and compost are 1.64 and 1.72 g/cm3, respectively*.

Table 8 provides the underlying data for characteristics of the leachate from soils in each of the three replicate pots in a given treatment such as leachate volume, leachate pH, and concentration of selected metals, simply reported as mean and standard deviation in the companion article [1]. Further, the underlying spectra and diffractograms for the proton nuclear magnetic resonance (^1^H-NMR) and X-ray diffraction (XRD) data, respectively, shown in figures and tables in the companion article are presented in Fig. 1, Fig. 2, Fig. 3, Fig. 4, Fig. 5, Fig. 6, Fig. 7. More specifically, the screenshots showing the NMR spectra contain information on the residuals (data-fit) and data error range, as well as the residual and data noise statistics, which indicate the accuracy of NMR measurements but is rarely published. These data show changes in the pore structure characteristics and mineralogical composition of the soil, soil-fertilizer, soil-biosludge, and soil-compost mixtures tested over time. Additional data on the evolution of the bulk and particle densities of the soil due to the amendments is shown in Fig. 8. It should be noted that the elemental composition of the soil in the different treatments is detailed in the companion article [1]. The dataset described here demonstrate the influence of the industrial biosludge on arid soil properties and alfalfa yields.

**Table 8:** Characteristics of the leachate from soils in each of the three replicate pots in a given treatment.

Treatment	Sample	Volume	pH	Total N	NO_3_^−^	PO_4_^3-^	SO_4_^2-^	Zn	Fe	K	Mn	Na	P
Soil (C1)	Rep. I	127.21	7.61	12.52	10.00	10.00	5001.20	0.20	0.72	184.27	0.27	5226.17	0.20
	Rep. 2	84.21	7.52	7.72	10.00	10.00	4343.75	0.22	0.65	158.12	0.53	4333.33	0.20
	Rep. 3	91.64	7.52	5.63	28.20	29.40	3803.67	0.28	0.29	165.67	0.25	3946	0.71
	Average	101.02	7.55	8.62	16.07	16.47	4382.87	0.23	0.55	169.35	0.35	4501.83	0.37
Soil + NPK + Urea (C2)	Rep. I	336.43	7.22	662.84	3024.50	10.00	3287.60	0.24	0.49	302.85	0.26	3629.20	0.86
	Rep. 2	300.29	7.21	1036.28	4206.45	10.00	3488.00	0.23	0.43	284.31	0.22	3713.09	1.19
	Rep. 3	386.79	7.15	501.75	2467.27	10.00	3111.73	0.20	0.49	196.59	0.23	3299.91	1.11
	Average	341.17	7.19	733.62	3232.74	10.00	3295.78	0.22	0.47	261.25	0.24	3547.40	1.06
Soil + 3% C (C3)	Rep. I	259.00	7.41	96.53	72.29	10.00	4812.56	0.21	0.51	1585.78	0.41	4359.78	1.11
	Rep. 2	359.71	7.43	112.68	202.57	10.00	4956.44	0.24	0.62	1640.30	0.68	4550.80	1.31
	Rep. 3	332.29	7.47	76.46	69.50	10.00	5004.71	0.26	0.37	1518.88	0.58	3835.63	1.15
	Average	317.00	7.44	95.22	114.79	10.00	4924.57	0.24	0.50	1581.65	0.56	4248.73	1.19
Soil + 0.75% BS (E1)	Rep. I	7.00	7.75	47.87	149.50	10.00	3820.50	0.20	0.50	153.00	0.20	3428.00	1.10
	Rep. 2	194.43	7.37	244.30	729.60	10.00	4158.40	0.28	0.63	211.48	0.22	4573.20	0.56
	Rep. 3	135.86	7.34	91.74	198.17	10.00	3721.83	0.54	0.32	403.43	0.32	3635.57	0.54
	Average	112.43	7.49	127.97	359.09	10.00	3900.24	0.34	0.48	255.97	0.25	3878.92	0.73
Soil + 1.5% BS (E2)	Rep. I	317.29	7.23	382.40	1717.86	10.00	4092.75	0.87	0.62	233.89	0.37	4749.13	0.65
	Rep. 2	276.79	7.21	122.91	1024.50	10.00	3935.29	0.78	0.77	252.21	0.47	4675.75	0.61
	Rep. 3	309.50	7.36	113.38	726.00	10.00	4245.71	1.03	0.60	179.84	0.32	4577.71	0.70
	Average	301.19	7.27	206.23	1156.12	10.00	4091.25	0.89	0.66	221.98	0.39	4667.53	0.65
Soil + 3% BS (E3)	Rep. I	173.07	7.14	547.97	2875.33	10.00	3723.78	0.85	0.47	326.44	0.38	5043.90	0.99
	Rep. 2	186.07	7.25	829.69	3384.88	10.00	3896.25	0.23	0.67	278.39	0.35	4524.25	0.80
	Rep. 3	279.57	7.22	532.77	2503.84	7.50	2670.27	0.22	0.58	216.10	0.24	2880.16	0.62
	Average	212.90	7.20	636.81	2921.35	9.17	3430.10	0.43	0.57	273.64	0.32	4149.44	0.81
Soil + 6% BS (E4)	Rep. I	374.50	7.02	958.58	4478.14	10.00	3035.25	0.39	0.83	337.45	0.39	4047.30	0.56
	Rep. 2	214.14	7.17	498.32	4302.33	10.00	3095.56	0.23	4.38	332.59	0.33	3704.20	0.44
	Rep. 3	357.93	7.04	978.68	3699.56	10.00	3187.67	0.57	0.95	293.48	0.37	4025.89	0.47
	Average	315.52	7.08	811.86	4160.01	10.00	3106.16	0.40	2.05	321.17	0.37	3925.80	0.49
Soil + 12% BS (E5)	Rep. I	392.36	7.72	2551.00	10.00	10.00	1553.60	0.36	9.01	552.56	0.26	4661.73	5.64
	Rep. 2	333.43	7.62	2062.86	10.00	10.00	2231.60	0.24	2.90	514.73	0.19	4155.13	7.53
	Rep. 3	67.79	7.91	1877.51	7.14	7.14	1314.25	0.22	3.31	434.93	0.23	3230.80	6.66
	Average	264.52	7.75	2163.79	9.05	9.05	1699.82	0.27	5.07	500.74	0.23	4015.89	6.61

In this and all subsequent tables, the dashes (−) indicate the absence of data. Thus, all treatments except E1 did not indicate flowering by the first cut.

## 2 Experimental design, materials, and methods

The materials and experimental methodology employed in this work are detailed in the companion article [1]. However, pertinent information is presented here to provide a complete description of how the dataset in this article were acquired.

### 2.1 Experimental materials

Cylindrical pots, 92 cm long and 52 cm in diameter, with a valve connected at the bottom to permit leachate collection were employed for the experiment. Leachate collection from the bottom of the pot was facilitated by using gravel (>2 mm) and fine sand in the bottom layer, which served to avoid clogging and facilitate water movement. A slope of 6–7° was created at the bottom of the pot by filling it with glass-reinforced plastic at a slight tilt to enable the direction of the leachate to the water collection valve.

A typical soil available in farms in Qatar was used as control (C1) for the experiments. It was obtained from the research experimental farm of the Agricultural Department of Qatar Ministry of Municipality and Environment at Rawdat Al-Faras, Al Khor. A commercially available 20-20-20 NPK fertilizer was used together with Urea in the second control treatment (C2) as detailed in Table 1 in Kogbara et al., [1]. The fertilizer was applied in three doses at 2, 12 and 24 weeks after planting. A commercially available compost, which corresponds to the type usually used in the farm was employed for the third control (C3) treatment. GTL biosludge with 90–95% dry solids obtained from a GTL plant in Qatar was used in the experiments. The pots were filled with samples of soil, and mixtures of soil, and inorganic fertilizer, 3% compost or 0.75–12% biosludge according to the details presented in Table 1 in Kogbara et al., [1]. The inorganic fertilizer (C2) and compost (C3) controls were used for comparison with the biosludge treatments (E1 – E5) to assess soil fertility improvement caused by biosludge amendment in contrast to typical fertilizer and compost application levels on farmlands in Qatar. Each treatment had three replicate pots arranged in a completely randomized design containing alfalfa seedlings.

### 2.2 Seeding, irrigation and sampling

The pots were first irrigated to set the soil columns before sowing of alfalfa seeds at 1 cm depth at 10 locations for each pot. Irrigation was applied to each pot manually every three days during the winter and daily during the summer. The amount of water applied was based on the irrigation water requirements of alfalfa for different months, which has an annual average of 2.71 mm/day, the lowest being 1.3 mm/day in January and the highest 5.6 mm/day in July. This was conducted to be in line with the normal irrigation practice of the Qatar Ministry of Municipality and Environment.

Soil samples were collected from the pots for initial analysis before seed sowing and at the final-growth stage (12 months) using a tube sampler (auger). The samples were collected from the top (top 20 cm depth) and bottom (remaining depth) portions of the pots at the final-growth stage for evaluation of the spatial variability of selected parameters (for e.g., porosity and density). Plant samples were collected after each cut (harvest). All pots were checked simultaneously for leachate formation every 2–4 weeks. The entire leachate volume drainable via the collection valve of the pots was collected in clean glass bottles during each sampling whenever leachate formation occurred.

### 2.3 Testing methods

The following is a description of the methods used for the analysis of the plant and leachate samples as well as the soil, soil-fertilizer, soil-biosludge, and soil-compost mixtures. For simplicity, mixtures of soil and other planting materials (fertilizer, compost, and biosludge) are referred to as soil in this section.

*Aboveground fresh biomass weight*: A stainless steel grass shear was used to collect samples for biomass determination from ten plants by snipping the plants at about 5 cm above ground level during each cut [2]. The fresh biomass weight was then taken. Three cuts were carried out on the plants at 3, 6 and 7 months after planting in line with the normal agronomic practice in Qatar.

*Plant height, number of tillers and days to flowering*: The plant height was determined by measuring the distance from the soil level to the terminal bud of the longest stem on that plant [3]. The number of main tillers/branches was determined by counting them from three randomly selected plants. The days to flowering was recorded as the number of days from the planting date to the opening of the first flower.

*Plant elemental content*: The elemental content of the plants was determined to evaluate the potential accumulation of elements from the biosludge in plant tissues. Biomass from plant cuts were dried and ground, and subjected to wet digestion with nitric acid. Thereafter, elemental content analysis was carried out using an iCAP 6000 Series ICP-OES (*Thermo Scientific*, USA).

*Leachate characteristics*: Leachates collected from the pots were filtered using 0.45-μm syringe cartridge filters to eliminate solid particles. A Mettler Toledo SevenMulti dual (conductivity/pH) meter was used to measure the pH of leachate samples. The leachate samples were subjected to ion chromatography (IC) following ASTM D 4327 [4] using an 850 Professional IC (*Metrohm*, Switzerland) for analysis of key anions (e.g. NO_3_^−^, PO_4_^3−^ and SO_4_^2−^). The aforementioned ICP-OES instrument was employed for analysis of metals in leachate samples after dilution with a 2% nitric acid solution following ASTM UOP714 [5]. The total nitrogen (Total N) content of the leachate samples was analyzed using a TOC-L series total organic carbon analyzer (Shimadzu, Japan) in line with the APHA Method 5310 [6].

*Pore structure characteristics*: Pore structure characteristics such as porosity and pore size distribution, which are among parameters that affect the flow of water through porous media [7], were characterized using a 2 MHz nuclear magnetic resonance (NMR) rock core analyzer with a 54 mm probe (*Magritek*, New Zealand). The equipment generates radio frequency signals or echoes from a saturated sample placed in a magnetic field. The initial amplitude of the signal indicates the total amount of fluid in the specimen, which in combination with a known volume of the saturation fluid is used to determine the cumulative or total porosity. The signal amplitude decays away with one or more relaxation times (T_2_) that are characteristic of the fluid and its environment. The relaxation time distribution gives information about the environment of the fluid, such as the pore size distribution in the sample. The T_2_ relaxation data was determined on a water-saturated soil sample placed in a 20-ml cylindrical plastic container. The Carr-Purcell-Meiboom-Gill (CPMG) sequence was used with 100 μs echo time, an inter-experimental delay time of 6500 ms and 200 scans. A Lawson and Hanson non-negative least squares fit method was then employed to analyze the CPMG decay using Prospa software (*Magritek*, New Zealand). The software also outputs the T_2_ log-mean, which is a proxy for the mean pore size. Details of the NMR technique are provided in Kogbara et al. [8].

*Soil mineralogical composition*: The mineralogical composition (crystalline minerals/phases) of the soil samples was monitored using X-ray diffraction (XRD) analysis. The analysis was conducted using a Rigaku Ultima IV multipurpose X-ray diffractometer (*Rigaku Corporation*, Tokyo, Japan). XRD pattern was collected at 2theta (2θ) angle from 3 to 80° with a step size of 0.01° and scanning speed of 0.5°/min. The XRD pattern was analyzed using the integrated Rigaku PDXL2 powder diffraction software.

*Dry bulk and particle densities*: The bulk density is important as it affects water and solute movement in the soil, and soil aeration. The particle density indicates the relative amounts of organic matter and mineral particles in a soil sample. The dry bulk density was determined as the ratio of the oven-dry (105 °C) weight of the soil to the total volume. The particle density was determined using the density bottle method as the ratio of the oven-dry soil weight to the volume of soil solids [9].
